# Three-Dimensional Evaluation of Soft Tissue Changes after Functional Therapy

**DOI:** 10.1155/2021/9928101

**Published:** 2021-04-24

**Authors:** Ersin Yıldırım, Şeniz Karaçay, Dilek Tekin

**Affiliations:** University of Health Sciences, Faculty of Dentistry, Department of Orthodontics, Istanbul, Turkey

## Abstract

This study was aimed at proposing a three-dimensional (3D) evaluation method for the soft tissue effects of Twin Block (TB) functional appliance therapy by using cone beam computed tomography (CBCT) images. In this retrospective study, a total of 60 pre- and posttreatment (T0 and T1) CBCT images of Class II patients with mandibular retrognathia treated with a TB appliance were used. Volumetric and linear soft tissue changes were evaluated quantitatively with 3D measurements and qualitatively with color mapping visual. Linear (NV-A and NV-Pog) and angular (SNA, SNB, and ANB) skeletal changes were also measured on 3D images. The Wilcoxon signed-rank test was used to compare statistical differences, and the scores of male and female participant differences were observed with the Mann–Whitney *U* test. In this study, a decrease was observed in SNA (*p* < 0.05), ANB (*p* < 0.01), and NV-Pog (*p* < 0.05) while an increase in SNB (*p* < 0.01) was found. However, decrease in NV-A distance was not statistically significant (*p* > 0.05). As a result of the evaluation of soft tissue changes, while the anterior reposition of the lower lip, soft tissue pogonion, and soft tissue gnathion was found to be significant (*p* < 0.01, *p* < 0.01, and *p* < 0.05, respectively), the upper lip and subnasale repositions were not statistically significant (*p* > 0.05). 3D soft tissue changes after TB therapy can be evaluated quantitatively and qualitatively by using CBCT images. Anterior repositioning of the mandible with functional therapy also provides improvement in soft tissue profile, especially in the lower facial region.

## 1. Introduction

One of the most important expectations of orthodontic patients with skeletal malocclusion is improvement in facial aesthetic appearance. Skeletal Class II malocclusions, which can affect facial aesthetics and reported to be present in approximately one-third of the population, are considered as one of the most common orthodontic anomalies [1]. Skeletal Class II malocclusions can be caused by maxillary prognathia, mandibular retrognathia, or a combination of both [2]. Although there are different treatment options depending on the characteristics of the case [3, 4], Twin Block (TB) is the most popular functional appliance frequently preferred in the treatment of patients with skeletal Class II malocclusion caused by mandibular retrognathia during the growth and development period [5, 6]. This appliance was developed by William J. Clark [7] to stimulate and increase mandibular growth by jumping the mandible anteriorly via two separate removable acrylic appliances constructed for maxilla and mandible which were including inclined planes. As the demand for facial attractiveness increases, soft tissue changes that will improve the facial profile become more important. Following the treatment of mandibular retrognathia with TB therapy, soft tissue adaptation can be seen in the lower facial region. Previous studies have also shown that the TB appliance improves the facial profile aesthetically [8].

Although there is a consensus in most of the studies on the skeletal and dentoalveolar effects of functional appliances [6, 9–12], there are different opinions on the reflection of these changes on the facial profile. In previous studies examining skeletal, dental, and soft tissue effects of the TB appliance on lateral cephalograms, it has been reported that maxillomandibular discrepancy and overjet are reduced, and there is advancement of the lower lip and pogonion as a result of stimulation of mandibular growth [10–12]. However, some studies have reported that there is no improvement in facial profile in every patient as a result of Class II functional appliance treatment, and individual differences can be seen [13–15].

There are many studies about TB and other functional appliances in the literature. However, it is observed that treatment success is generally expressed only by hard tissue changes [6, 9, 10, 12], and the reflection of treatment on soft tissue is usually neglected. Studies examining soft tissue changes have generally been carried out on two-dimensional (2D) lateral cephalograms [8, 15–17]. In studies examining 3D soft tissue changes, measurements were also made on 2D cephalograms. In addition to these measurements, 3D surface images obtained by methods such as optical surface laser scanning and stereophotogrammetry were used, and evaluation of soft tissue profile alterations like advancement of the lower lip and chin was determined by the “color mapping” method [18–22]. To date, no study has investigated the soft tissue changes resulting from Class II TB treatment by measurements made on CBCT images. Thus, this retrospective study is aimed at examining and proposing a 3D evaluation method for the soft tissue effects of TB functional appliance therapy by using CBCT.

## 2. Materials and Methods

### 2.1. Subjects

In this retrospective study, a total of 60 pre- and posttreatment CBCT images that had been obtained previously for 3D evaluation of posterior airway space changes of Class II patients with mandibular retrognathia treated with a TB appliance were used. These images of a total of 30 patients (16 males and 14 females; mean age 12.83 ± 1.17 and 12.50 ± 1.23 years, respectively) were obtained after the approval of the Ethics Committee of Gulhane Military Medical Academy for this study (1491-107-11/1539-1588).

All images belong to patients with the following criteria:

- Skeletal Class II malocclusion with normal maxilla and mandibular retrognathia (ANB angle at least 5°)
- Dental bilateral (molar and canine) Class II relation and minimal or no crowding in both arches
- Increased overjet (at least 5 mm)
- Normal growth pattern or horizontal and have growth potential according to cervical vertebra maturation indicators
- Without any syndromes and craniofacial anomalies

TB appliance was used in all patients until Class I canine and molar relationship was achieved and the overjet was eliminated. The average time between pretreatment (T0) and postfunctional treatment (T1) CBCT images was 7.4 months. Facial soft tissues changes were evaluated on CBCT images by using quantitative and qualitative manner.

### 2.2. Data Collection and Measurements

All of the CBCT images were obtained with the same device (ILUMA IMTEC, 3M Company, Ardmore, Oklahoma, USA) with the patient in a sitting position, the Frankfurt plane parallel to the ground and maximum intercuspation in centric occlusion. The device performed high-resolution scanning at 3.8 mA and 120 kV, 360-degree unidirectional, and with a rotation of 20 seconds; images with a field of view (FOV) of 21.1 × 14.2 cm and an isotropic voxel size of 0.290 mm were generated. Images in Digital Imaging and Communications in Medicine (DICOM) file format were transferred to SimPlant Master Crystal v13 (Materialize Dental, Leuven, Belgium) image processing software, and landmark determination, measurement, and registration on 3D images were made using the same software.

The skeletal changes were compared by angular (SNA, SNB, and ANB angles) and linear (NV-A and NV-Pog distances) measurements using the 3D cephalometry function under the OMS module of the image processing software.

**Figure 1:**
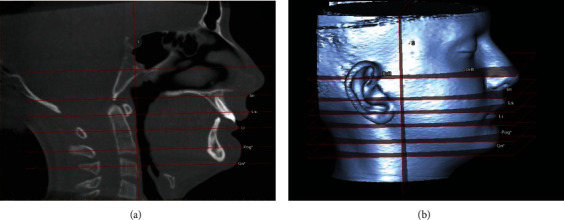
Reference planes generated on the 3D images to evaluate soft tissue changes.

To perform the superimpositions and to measure the changes, 3D soft tissue images were obtained by segmentation on T0 and T1 images. For the standardization of the segmentation process, predetermined soft tissue thresholding values in the SimPlant software (min: -700, max: 225 Hounsfield units) were used. Seven reference planes were formed on the 3D images for soft tissue measurements (Figure 1):

- FH (Frankfort horizontal) plane: the plane formed by right *porion* (Po) and left and right *orbitale* (Or) points
- Sella vertical (SV) plane: the plane passing through the *sella* (S) point and perpendicular to the FH plane
- Subnasale (Sn) plane: the plane parallel to the FH plane, perpendicular to the SV plane, and passing through the *subnasale* (Sn) point
- Upper lip (Ls) plane: the plane parallel to the FH plane, perpendicular to the SV plane, and passing through the *labrale superius* (Ls) point
- Lower lip (Li) plane: the plane parallel to the FH plane, perpendicular to the SV plane, and passing through the *labrale inferius* (Li) point
- Soft tissue pogonion (Pog′) plane: the plane parallel to the FH plane, perpendicular to the SV plane, and passing through the *soft tissue pogonion* (Pog′) point
- Soft tissue gnathion (Gn′) plane: the plane parallel to the FH plane, perpendicular to the SV plane, and passing through the *soft tissue gnathion* (Gn′) point

**Figure 2:**
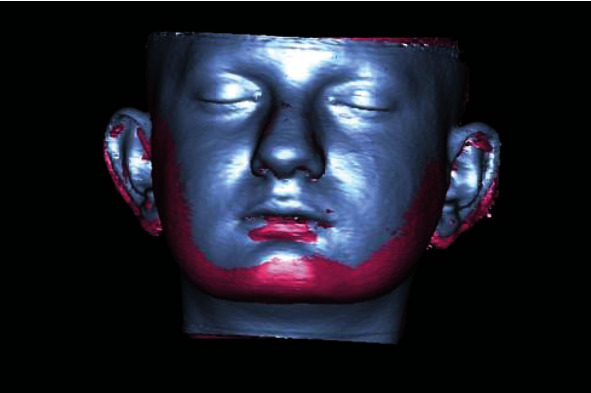
Superimposition of the T0 (blue area) and T1 (pink area) 3D images.

**Figure 3:**
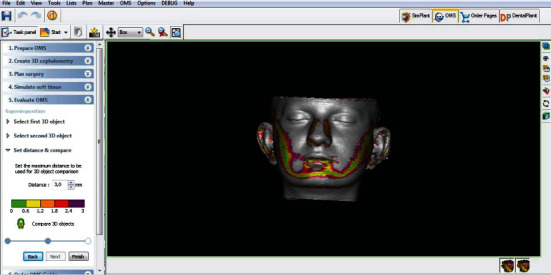
Color-coded map visualization of the 3D soft tissue changes.

Linear measurements between the 5 soft tissue landmarks (Sn, Ls, Li, Pog′, and Gn′) and SV plane were performed on T0 and T1 images separately. The localization of each landmark used for all measurements was determined by checking in three dimensions of CBCT images. All measurements were carried out by the same researcher (EY). The software's “superimposition” function based on a rigid body registration was used for superimposition of T0 and T1 3D soft tissue images (Figure 2). Thereafter, soft tissue changes in each patient could be visualized using a color-coded map (Figure 3). Volumetric and linear changes in the soft tissue were evaluated quantitatively with 3D measurements and qualitatively with color mapping visual.

### 2.3. Statistics

A power analysis established by G∗Power software (v3.1.3; Franz Faul, Universität Kiel, Germany) revealed that the sample size of 30 patients provided more than 80% power to detect significant differences with an effect size of 0.50 between the two measurements at a 0.05 significance level. Randomly selected 10 images were remeasured 2 weeks later by the same investigator, and the Cronbach alpha reliability analysis result was found to be high (0.925).

MS Excel (Microsoft, Seattle, Washington, USA) and Gnu PSPP (Free Software Foundation, Inc. http://www.gnu.org/software/pspp/get.html) programs were used for all statistical analysis and calculations.

The Kolmogorov-Smirnov normality test (with the Lilliefors significance correction) and Levene's variance homogeneity test were applied to the data. The mean measurement values and standard deviations (SD) were calculated for T0 and T1. The Wilcoxon signed-rank test was used to compare T0 and T1 scores, and the Mann–Whitney *U* test was used to compare the scores of male and female participants.

## 3. Results

Mean values and standard deviations of soft tissue and skeletal measurements and comparison for T0 and T1 are given in Table 1.

Evaluation of skeletal alterations revealed that the SNA angle decreased (*p* < 0.05) and the SNB angle increased (*p* < 0.01). Depending on these changes, the ANB angle decreased significantly (*p* < 0.01). When we examine the anteroposterior alteration of the mandible and maxilla, there was a significant decrease in NV-Pog distance (*p* < 0.05) while the decrease in NV-A distance was not significant (*p* > 0.05). It appears that the maxilla is repositioned slightly in the posterior direction, while the mandible is repositioned significantly in the anterior direction due to the TB therapy.

In the evaluation of soft tissue changes, while the anterior reposition of the lower lip (SV-Li), soft tissue pogonion (SV-Pog′), and soft tissue gnathion (SV-Gn′) was found to be significant (*p* < 0.01, *p* < 0.01, and *p* < 0.05, respectively), the upper lip (SV-Ls) and subnasale (SV-Sn) repositions were statistically insignificant (*p* > 0.05). Differences by gender were insignificant for all measurements (*p* > 0.05).

## 4. Discussion

Mandibular retrognathism is considered to be a predominant factor for the development of Class II orthodontic malocclusions [23]. Therefore, mandibular advancement with functional appliances is a frequently preferred treatment approach during the growth and development period. TB is one of the most commonly preferred functional appliances in the treatment of skeletal Class II caused by retrognathic mandibula in growing children [6, 17, 23]. An important factor in choosing removable or fixed functional devices is the need for patient compliance because it is thought to have a significant effect on treatment results [24]. Since TB consists of two separate plates, it facilitates speech and jaw functions and therefore increases patient compliance. The first change noticed by orthodontic patients and their parents is the reflections of functional treatment on soft tissue. Rapid improvement in profile also increases cooperation [24].

Although there are many studies about the skeletal effects of functional treatments in the literature, it is observed that the success of the treatment is limited with the hard tissue changes by the investigators [1, 4–6, 9, 10, 13–15, 25]. Soft tissue effects of TB functional therapy were examined with different imaging methods and analysis systems previously. Quintão et al. [8] investigated the facial profile changes in addition to skeletal and dental changes in Class II patients treated with TB functional appliance compared with untreated controls. They revealed that the ANB angle decreased, and the mandibular length increased significantly. Although there was retraction in the upper lip and forward movement in the soft tissue pogonion, they reported that there was no change in the lower lip position compared to the aesthetic line (E-line). Singh and Clark [16], using a color-coded finite element analysis, found a decrease in prominence of the labiomental groove associated with TB treatment. Gulec and Goymen [15] also investigated soft tissue and skeletal changes in skeletal Class II patients treated with TB and Forsus fatigue-resistant device appliances compared with the untreated control group. While both devices stimulate mandibular growth and there was a significant decrease in the ANB angle, no significant change was found in soft tissue parameters (upper lip and lower lip to E-line) in their study. In the study of Baysal and Uysal [17], the effects of TB and Herbst appliances on soft tissue were examined and the results were compared with those of the untreated controls. They revealed that the SNA and ANB angles decreased and the SNB angle increased significantly, and more advancement of soft tissue pogonion and lower lip was observed in the TB group.

In the studies mentioned above, soft tissue effects of TB functional therapy were assessed by 2D lateral cephalograms. However, 2D imaging has several disadvantages, such as variation in magnification, projection, and superimposition and head position errors [26–30]. Furthermore, significant differences were reported, especially in linear measurements [31, 32]. Therefore, current conventional anteroposterior and lateral cephalograms are insufficient for examining and measuring the 3D objects exactly.

There are few studies examining 3D facial soft tissue change after functional treatment [18–22]. However, in these studies, besides 3D surface images, 2D cephalograms were used for soft tissue measurements. To date, no study has investigated the soft tissue changes resulting from Class II TB treatment by measurements made only on 3D images. Morris et al. [22] evaluated the soft tissue effects of functional appliances (Bionator, Bass, and TB) using lateral cephalometry and optical surface laser scanning. However, soft tissue changes were examined by measurements made on 2D cephalograms. The 3D laser scan images were superimposed with the control group images and evaluated visually. As a result of 2D cephalometric analysis, they stated that there was an increase in the chin protrusion and the forward movement in the lower lip compared to the E-line in the TB group. McDonagh et al. [18], Sharma and Lee [19], and Lee et al. [21] also used lateral cephalometry and optical surface laser scanning methods in the examination of the soft tissue changes after TB and other functional appliances (Bass, TB, and TB+high-pull headgear; TB and Dynamax; and TB and miniblock (MB), respectively). In all three studies, anteroposterior soft tissue changes were also measured using 2D cephalometry. Pretreatment and posttreatment superimposed 3D laser surface scan images were compared with millimetric color mapping scale. 2D cephalometric analysis indicated that forward movement in the soft tissue pogonion was greater significantly, and there was not significant forward movement in the lower lip according to the E-line in the TB group. Salloum et al. [20] compared pretreatment stereophotogrammetric images with the construction bite and posttreatment 3D images for estimating and evaluating facial soft tissue changes after modified TB therapy. They also used pre- and posttreatment 2D cephalograms for angular and linear measurements.

In the present study, we aimed to examine and to propose an exact 3D evaluation method for the soft tissue effects of TB functional appliance therapy by using CBCT. The CBCT images allow clinically accurate and reliable 3D measurements when using the appropriate voxel size and method. Head orientation during the CBCT scan does not affect the accuracy of linear measurements [33, 34]. Radiation doses of contemporary CBCT have been reported to be significantly lower than conventional medical CT and have equivalent radiation doses to conventional imaging modalities such as full mouth series of intraoral radiographs [35]. In the present study, additional cephalometric or panoramic radiographs were not taken to prevent unnecessary dose exposure, as low as diagnostically acceptable CBCT images were used for cephalometric analysis.

In our study, the SNA angle decreased and the SNB angle increased after the TB therapy. Depending on these changes, the ANB angle also decreased significantly. There was a significant decrease in NV-Pog distance due to the forward mandibular displacement. It appears that the maxilla is repositioned slightly in the posterior direction, while the mandible is repositioned significantly in the anterior direction. Similar to our results, the efficiency of TB in forward mandibular reposition [5, 6, 15, 17] and restraining effect on the maxillary growth [6, 17] has been demonstrated in different studies.

It appears that different measurements are used in some study to evaluate soft tissue changes after TB treatment. However, some measurements made using anatomical structures that are likely to change because of treatment should be carefully evaluated. For example, in most of the studies, the E-line was used to evaluate the lower and upper lip positions [8, 15, 18, 19, 21, 22]. The E-line is not a good reference plane to evaluate lip position alterations because of the changes that may occur in the pronasale and soft tissue pogonion points due to growth or treatment effects. This may be the reason why in most of these studies no significant forward movement was detected in the lower lip after TB therapy. In the present study, lip positions were evaluated according to the reference plane (SV) that would not be affected by the treatment. Results of the present study support some of the abovementioned study's findings such as stabile upper lip position after TB therapy, while most significant changes occurred in the lower facial region. In particular, in the lower lip, soft tissue pogonion, and soft tissue gnathion points, forward movement was determined quantitatively and qualitatively.

In the present study, we aimed to determine the pure and immediate effects of TB therapy on the soft tissue independent from any other orthodontic fixed treatment effects. In our study, the average treatment duration was 7.4 months. The absence of the untreated skeletal Class II control group can be considered as the limitation of the study. However, Morris et al. [22] found no significant facial soft tissue growth in control subjects during the 9-month study period. Quintão et al. [8] also observed only minor facial changes in the control group during the 12-month study period. Considering the short duration of the present study, it can be concluded that the soft tissue changes were independent from the natural growth.

## 5. Conclusion

- CBCT is an effective technique for quantitative and qualitative evaluation of soft tissue changes after functional treatment
- After functional treatment with TB, the most important soft tissue changes occurred in the lower facial region, especially in the lower lip, soft tissue pogonion, and soft tissue gnathion points, as forward movement

## Figures and Tables

**Table 1:** Descriptive statistics of the measurements with results of the Wilcoxon signed-rank tests.

Measurements	T0		T1		*p*
	Mean	SD	Mean	SD	
SNA (°)	81.65	1.61	81.33	1.48	0.028^∗^
SNB (°)	74.63	1.44	78.36	1.53	0.005^∗∗^
ANB (°)	7.02	0.96	2.97	0.79	0.005^∗∗^
NV-A (mm)	1.86	0.42	1.54	0.46	0.057
NV-Pog (mm)	-8.36	1.93	-4.78	1.88	0.022^∗^
SV-Sn (mm)	86.35	4.27	87.02	4.14	0.189
SV-Ls (mm)	88.68	5.36	88.24	5.72	0.084
SV-Li (mm)	83.24	5.89	85.31	6.31	0.005^∗∗^
SV-Pog′ (mm)	74.99	6.22	77.13	7.31	0.005^∗∗^
SV-Gn′ (mm)	72.58	5.99	75.12	4.03	0.014^∗^

SD: standard deviation. ^∗^*p* < 0.05; ^∗∗^*p* < 0.01.

## Data Availability

The data used to support the findings of this study are included within the article.
